# Serum Levels of CA125 and HE4 as a Tool for Predicting Regional Lymph Node Metastatic Involvement in Endometrial Carcinoma

**DOI:** 10.3390/cancers17172740

**Published:** 2025-08-23

**Authors:** Tomáš Crha, Petra Ovesná, Vít Weinberger, Michal Felsinger, Branislav Babjak, Dalibor Valík, Jitka Hausnerová, Luboš Minář

**Affiliations:** 1 Department of Gynecology and Obstetrics, University Hospital Brno and Faculty of Medicine, Masaryk University, 60200 Brno, Czech Republic; crha.tomas@fnbrno.cz (T.C.); weinberger.vit@fnbrno.cz (V.W.); felsinger.michal@fnbrno.cz (M.F.); babjak.branislav@fnbrno.cz (B.B.); 2 Institute of Biostatistics and Analyses, Faculty of Medicine, Masaryk University, 62500 Brno, Czech Republic; ovesna@iba.muni.cz; 3 Department of Laboratory Medicine, Masaryk University and University Hospital Brno, 62500 Brno, Czech Republic; valik.dalibor@fnbrno.cz; 4 Department of Laboratory Methods, Masaryk University, 62500 Brno, Czech Republic; 5 Department of Pathology, University Hospital Brno and Faculty of Medicine, Masaryk University, 60200 Brno, Czech Republic; hausnerova.jitka@fnbrno.cz

**Keywords:** endometrial carcinoma, lymph node metastasis, nodal staging, tumour markers, CA125, HE4

## Abstract

**Background**: Endometrial carcinoma is the most common gynaecological malignant tumour in developed countries. At present, no routinely used serum biomarker is available for the prediction of lymph node metastasis (LNM). This study thus evaluates the potential of tumour markers CA125 and HE4 as LNM predictors in endometrial carcinoma patients. **Objectives**: The aim of this study was to evaluate the potential use of CA125 and HE4 and to assess the viability of a model developed using the parameters of serum tumour marker levels for LNM risk stratification. **Methods**: A retrospective, single-institution study of 220 patients with biopsy-proven endometrial carcinoma was conducted from May 2020 to December 2023. Preoperative serum levels of HE4 and CA125 were determined. All patients underwent surgical lymph node staging. The study evaluated the sensitivity and specificity of tumour markers and of the developed LNM risk prediction model. **Results**: No LNM was observed in 167 of the 220 patients (75.9%), micrometastatic lymph node involvement was observed in 13 patients (5.9%), and macrometastatic involvement was observed in 24 patients (10.9%). Median CA125 and HE4 levels were significantly higher in patients with LNM than in those without. With a CA125 cut-off value of 35 IU/mL, a sensitivity of 70% and a specificity of 92% were obtained, while an HE4 cut-off value of 103 pmol/L yielded a sensitivity of 78% and a specificity of 80%. A prediction model combining CA125, HE4, and the extent of uterine invasion, as detected by ultrasound, yielded a sensitivity of 84% and a specificity of 98% in predicting LNM. **Conclusions**: CA125 and HE4, along with the prediction model, facilitate endometrial carcinoma patient subdivision into low- and high-risk LNM groups. As this method is technically simple, non-invasive, and inexpensive, it could be of undeniable benefit in the risk stratification of patients with multiple comorbidities, which limit the duration and extent of surgery.

## Simple Summary

Endometrial carcinoma (EC) is the most common gynaecological malignant tumour in middle- and high-income countries. Preoperative lymph node (LN) involvement is generally evaluated using imaging methods. Serum biomarkers are not routinely used for predicting lymph node metastasis (LNM). This retrospective study thus analyses the potential of serum levels of cancer antigen 125 (CA125) and human epididymis protein 4 (HE4) to predict the metastatic involvement of regional lymph nodes. The results suggest that CA125 and HE4 may be used to stratify patients into low- and high-risk LNM groups. Further refinement, which necessitates greater expenses as well as substantial gynaecological expertise, may be achieved using specific models, including, in this case, an ultrasound examination of the extent of uterine infiltration. Such approaches could be used for planning the extent of surgical treatment.

## 1. Introduction

Endometrial carcinoma (EC) is the most common gynaecological malignant tumour in Europe, with 124,874 new cases diagnosed in 2022 [1] and a five-year prevalence rate of 34.7% (445,805 cases) [2]. Due to early symptom onset, most cases are diagnosed at an early stage [3]. The five-year survival prognosis is 95% for stages 1 and 2, 68% for stage 3, and 17% for stage 4 [4]. Surgical treatment involving hysterectomy and bilateral adnexectomy is the standard of care. Lymph node (LN) staging is an essential part of surgery, as it allows for the assessment of the extent of the disease and the subsequent indication of adjuvant therapy. Sentinel lymph node biopsy (SNB) is the standard method of staging in all patients with presumed uterus-confined disease. If no sentinel lymph nodes are detected on either side of the pelvis, side-specific systematic lymphadenectomy should be performed for high-intermediate and high-risk patients and may be considered for presumed intermediate risk patients [5]. However, lymph node staging, and especially systemic lymph node dissection, increases perioperative (vascular and nerve injury, higher blood loss) and postoperative (lymphoedema, lymphocyst) morbidity and prolongs the duration of each operation as well as the duration of anaesthesia [6,7]. According to guidelines issued by the European Society of Gynaecological Oncology (ESGO), the European Society for Radiotherapy and Oncology (ESTRO) and the European Society of Pathology (ESP), the risk of lymph node metastasis (LNM) is preoperatively assessed using imaging methods, predominantly CT [5,8]. The sensitivity of imaging methods used for LNM detection, especially ultrasound and CT, is low [8,9,10,11]. As advanced and more precise imaging methods such as MRI and PET/CT are not always available, the need for non-invasive and economically viable diagnostic methods arises—especially such methods which could serve as a suitable supplement or replacement for the imaging techniques currently dominating the field. At present, no serum biomarker capable of predicting LNM with sufficient sensitivity is routinely used. We focus mainly on cancer antigen 125 (CA125) and human epididymis protein 4 (HE4). CA125 is a glycoprotein which is mainly used in the diagnosis and monitoring of ovarian carcinomas [12]. HE4 is a serum glycoprotein which is elevated in epithelial ovarian cancer (EOC), but also, e.g., in gastrointestinal and lung cancers [13,14]. Their role in the diagnosis, prognosis and monitoring of EC has been previously explored [15,16,17]. The biological plausibility of HE4 as a marker of LNM in EC is based on its overexpression in aggressive EC, its promotion of invasion, proliferation, and metastasis of tumor cells and possible regulation of proteolytic enzymes [17]. It is assumed that CA125 is a plausible marker thanks to its association with agressive histotypes. This association suggests that elevated serum CA125 may reflect the greater invasiveness and dissemination of a tumour which includes LNM [18,19]. Indeed, previous studies report a significant correlation between elevated serum HE4 and/or CA125 levels and LNM [15,18,20,21,22,23]. To date, only a limited number of studies have combined CA125, HE4, and imaging findings for preoperative EC nodal risk stratification, and very few have assessed their utility in a sizeable real-world cohort. This study aims to assess the individual and combined predictive ability of CA125 and HE4, alongside ultrasound-based uterine invasion assessment, for identifying regional LNM in EC patients. This approach could be used for planning the extent of surgical treatment. Specifically, in case of equivocal imaging findings, it could shorten surgery duration and reduce morbidity in patients with severe internal comorbidities, since those in the low-risk LNM group would not require any surgical LN staging (e.g., SNB or lymphadenectomy). Consequently, systematic pelvic lymphadenectomy could be avoided in cases of SNB failure. Moreover, this approach could contribute to shorter hospital stays and lower costs associated with the management of post-operative complications.

## 2. Materials and Methods

### 2.1. Study Cohort

This single-institution retrospective study, along with the associated informed consent, was approved by the Ethics Committee at University Hospital Brno, Czech Republic (No. 24-120624/EK). The study was conducted at our institution from May 2020 to December 2023 and included a total of 220 patients. No formal sample size calculation was performed prior to study initiation, as the study included all eligible patients treated at our institution during the defined period. All patients underwent an ultrasound examination to assess the extent of potential uterine infiltration prior to diagnostic intervention. Disease diagnosis was established by endometrial biopsy using either dilation and curettage or hysteroscopy. Serum tumour marker levels were determined from blood samples collected immediately prior to the diagnostic procedure. This approach was adopted to negate the influence of potential tumour volume reduction on the marker levels. The course of treatment was determined by a multidisciplinary board based on preoperative staging. Disease stage classification was performed in accordance with the 2009 classification system established by the International Federation of Gynaecology and Obstetrics [24]. The cohort comprised 7 premenopausal and 213 peri/postmenopausal patients.

All patients included in the study underwent a complete surgical staging procedure, which included hysterectomy with either bilateral salpingo-oophorectomy or bilateral salpingectomy in premenopausal patients who met the ovarian preservation criteria (a total of 7 patients). The surgical staging options for LN were as follows: bilateral sentinel node biopsy (SNB), bulky lymph node resection, systemic aorto-pelvic lymphadenectomy, unilateral SNB, and side-specific lymphadenectomy on the contralateral side in case of SNB failure on a given side. In line with current recommendations, the most common approach was SNB and, in the event of SNB failure in indicated cases, side-specific pelvic lymphadenectomy. When bulky nodes were detected, targeted resection was performed. Cases of systematic aortopelvic lymphadenectomy concern advanced FIGO stages with spread outside the uterus and serous histotype [5].

Inclusion criteria: patients diagnosed with endometrioid, serous, or mixed histotype carcinoma with an endometrioid component who had undergone complete surgical lymph node staging.

Exclusion criteria: missing, unsuccessful, or incomplete surgical lymph node staging, ambiguous histology, renal insufficiency—defined by estimate of glomerular filtration rate (GFR) lower than 60 mL/min/1.73 m^2^, as determined by checking creatinine levels and reviewing medical histories—additional malignancy, autoimmune disease, pelvic inflammation, or endometriosis. Molecular classifiers were not subjected to investigation. Additional investigated parameters included the extent of uterine invasion (limited to the endometrium, invasion of < ½ of myometrial thickness, invasion of ≥ ½ of myometrial thickness, cervical stromal invasion, uterine serosal invasion), disease stage according to FIGO 2009, age at diagnosis, obesity (BMI over 30), and the presence of hypertension, diabetes, or ischemic heart disease.

### 2.2. Examination of Tumour Markers

Blood draws to determine serum tumour marker levels were performed concurrently with the diagnostic procedure to eliminate the influence of tumour volume reduction on their plasma levels. All samples were drawn under fasting conditions and processed and analysed on the same day as real-time standard clinical specimens. Blood was drawn into plain tubes following standard laboratory procedures containing no anticoagulant and allowed to clot for one hour. The tubes were then centrifuged in the automated Roche pre-analytics module to obtain serum. Tumour markers CA125 and HE4 were analysed using an automated electrochemiluminiscent assay on the Cobas Roche 801 analyser according to the manufacturer’s instructions. Both methods are accredited according to the ISO 15189 standard. The analytical performance of both methods is as follows: the minimum detection level for CA125 was 1.2 kU/l, the intraassay coefficient of variation was 1.31%, the interassay coefficient of variation was 2.07% and reference ranges were 035 kU/l; the minimum detection level for HE4 was 15 pmol/l, the intraassay coefficient of variation was 2.4%, and the interassay coefficient of variation was 2.2%. Reference ranges for women younger/older than 50 years were 0–70 pmol/l and 140 pmol/l, respectively, reflecting pre/postmenopausal levels.

### 2.3. Histological Examination

During the course of surgery, the sentinel LNs were identified using indocyanine green (ICG). Following extraction, the LNs were fixed in 10% buffered neutral formalin and processed for histological examination. Each lymph node was typically cut into 2 mm lamellae, which were then stained with haematoxylin and eosin (H&E). In case the nodules were negative for metastases following the initial staining, they were subjected to ultrastaging. This method involves the creation of additional H&E sections and immunohistochemical staining for keratins (e.g., AE1/AE3) to facilitate the detection of small metastases. Through repeated slicing and periodic immunohistochemical section insertion, the node is thus processed in its entirety. All lymph nodes intraoperatively identified by the surgeon as sentinel LNs were processed using the ultrastaging protocol. Ultrastaging helps identify micrometastases (0.2–2 mm) and isolated tumour cells (ITC, ≤ 0.2 mm) according to the definitions provided in the AJCC Cancer Staging Manual, 8^th^ edition [25]. Non-sentinel LNs were generally processed whole and evaluated based on baseline H&E staining. Pathologists were blinded to biomarker values.

### 2.4. Data Analysis

Basic descriptive characteristics were used for summarisation, including absolute and relative frequencies for categorical data, and the mean, standard deviation (SD), median, and interquartile range (IQR) for continuous variables. Comparisons were performed using Fisher’s exact test, the Mann–Whitney test (2 groups), the Chi-square test, or the Kruskal–Wallis test (more than 2 groups). ROC analysis was used to determine the cut-off for HE4 and CA125 markers with the highest Youden’s index (i.e., the maximum sum of sensitivity and specificity) for predicting metastatic involvement. The diagnostic parameters of sensitivity, specificity, and predictive values were then calculated for these cut-off values. To assess the predictive ability of the markers in combination with other characteristics, decision trees were constructed, and performance parameters such as overall accuracy, sensitivity, specificity, and AUC were again calculated. A decision tree classifier was trained using 2-fold cross-validation, with a minimum split size of 12, complexity parameter (cp) of 0.02, and maximum tree depth of 5. No imputation methods were applied, as the dataset was complete with no missing values. All analyses were performed in R (v4.3.2) using pROC (v1.18.5), epiR (v2.0.70), and rpart (v4.1.21) packages. All tests were two-tailed with a significance level of 5%; no multiple comparisons correction was applied.

## 3. Results

Of the 220 evaluated endometrial carcinoma (EC) patients, a majority (159, 72.3%) were diagnosed with low-grade (LG) endometrioid carcinoma. High-grade (HG) endometrioid carcinoma was present in 34 patients (15.5%), serous carcinoma in 16 patients (7.3%), and mixed carcinoma with an endometrioid component in 11 patients (5.0%). No LNM was observed in a total of 167 patients (75.9%) while micrometastatic involvement was observed in 13 patients (5.9%) and macrometastatic involvement in 24 patients (10.9%). ITCs were present in 16 patients (7.3%). With respect to the type of surgical LN staging, bilateral SNB was successful in 158 patients (71.8%), systemic aorto-pelvic lymphadenectomy was performed in 53 patients (24.1%), unilateral SNB and systemic contralateral pelvic lymphadenectomy was performed in 6 patients (2.7%), and bulky lymph node resection was performed in 3 patients (1.4%). In most cases, the disease was detected in the early stages: 119 patients (54.1%) were in FIGO stage IA, 28 (12.7%) in FIGO stage IB and 29 (13.2%) in FIGO stage II. A total of 44 patients (20%) were in the advanced stage of the disease. A definitive histopathological examination of the uterus revealed a myometrial invasion of <½ in 88 patients (40%), an invasion of ≥½ in 47 patients (21.4%), cervical stromal invasion in 41 patients (18.6%), and uterine serosal invasion in 9 patients (4.1%). The mean age of the entire study population was 64 years. 126 patients (57.3%) had a body mass index (BMI) over 30, 134 (60.9%) were diagnosed with hypertension, 49 (22.3%) with diabetes mellitus, and 15 (6.8%) with ischemic heart disease. The population characteristics are shown in Table 1.

**Figure 1:**
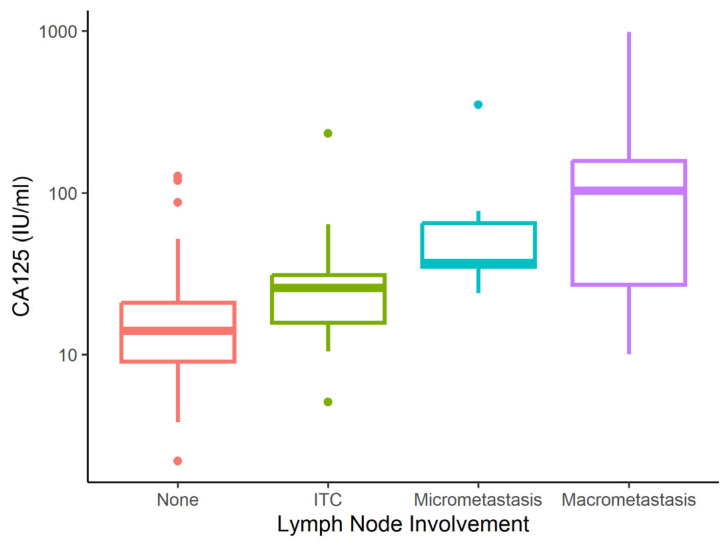
Cancer antigen 125 (CA125) level according to metastatic involvement. ITC = isolated tumour cells; None = no micrometastasis, macrometastasis or ITC.

**Figure 2:**
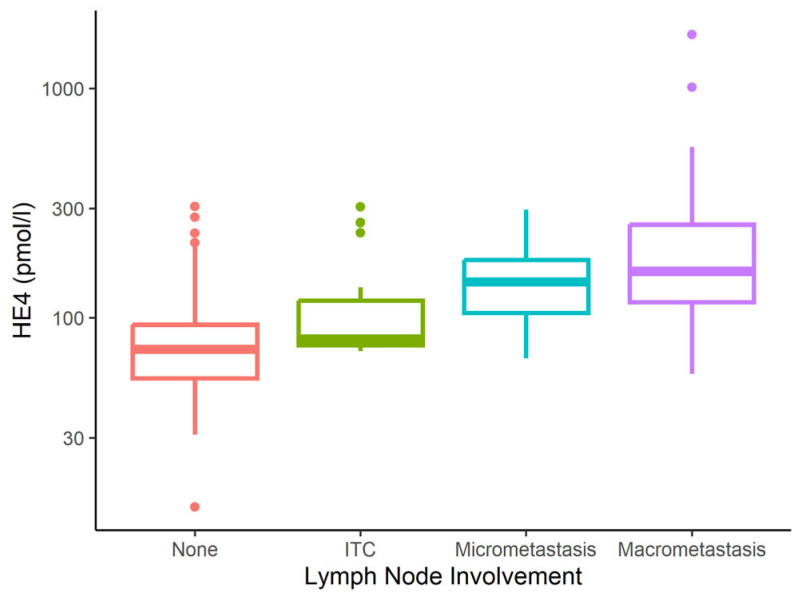
Human epididymis protein 4 (HE4) level according to metastatic involvement. ITC = isolated tumour cells; None = no micrometastasis, macrometastasis or ITC.

Table 2 shows serum CA125 and HE4 values in relation to clinical data. Statistical analysis revealed median CA125 and HE4 IQR levels of 14 (9–23) IU/mL and 74 (56–96) pmol/L, respectively, in the pooled group of patients with no metastatic involvement and patients with ITC+ findings. CA125 and HE4 levels were significantly higher in patients with LNM than in those without, including ITC+ findings. In the case of micrometastatic involvement, the median CA125 and HE4 levels were 37 (35–65) IU/mL and 144 (105–179) pmol/L, respectively. In the case of macrometastatic involvement, the median was 103 IU/mL (28–158) and 160 pmol/L (118–255) (see Figure 1 and Figure 2).

When serum levels were compared between two prognostically different cohorts (i.e., the micro/macrometastatic LN involvement group and the group without metastatic involvement, including ITC+), the analysis revealed a statistically significant difference (*p* < 0.001). The comorbidities investigated in this study did not appear to be statistically significant factors for LNM. In the case of ITC findings, the median CA125 and HE4 values were 26 (16–31) IU/mL (*p* = 0.005) and 81 (76–102) pmol/L (*p* = 0.004), respectively.

The population characteristics of the two baseline prognostically different groups with different treatment strategies are shown in Table 3.

A hypothetical assessment of the ITC+ finding as metastatic involvement (inconsistent with clinical practice) would yield median CA 125 and HE4 values of 14 (9–21) IU/mL and 73 (55–93) pmol/L in unaffected nodes, and 37 (24–105) IU/mL and 136 (79–224) pmol/L in metastatic nodes (*p* < 0.001).

### 3.1. CA125 and HE4 Sensitivity and Specificity for Determining Metastatic Involvement

A CA125 cut-off of 35 IU/mL yielded a sensitivity of 70% (95% CI: 53–84%) and a specificity of 92% (87–92%). The positive predictive value (PPV) for serum CA125 was 63% (47–78%) (95% CI: 4–78%) and the negative predictive value (NPV) was 94% (89–97%). The AUC for CA125 was 0.78 (0.7–0.87). A HE4 cut-off of 103 pmol/L yielded a sensitivity of 78% (95% CI: 62–90%) and a specificity of 80% (74–85%). The PPV for serum HE4 was 44% (95% CI: 32–57%) and the NPV was 95% (95% CI: 90–98%). The AUC for HE4 was 0.77 (0.7–0.85). These univariate models only take into account the predictive power of the markers for metastatic involvement while omitting additional patient characteristics.

### 3.2. LNM Prediction Model

**Figure 3:**
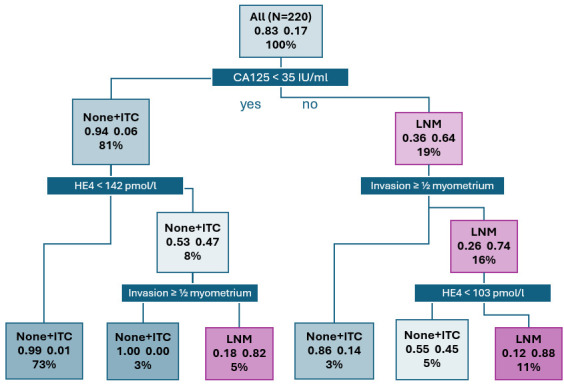
Lymph node metastasis (LNM) prediction model. Xx = actual proportion of patients with negative lymph nodes or LNM in each subgroup according to the combination of selected factors; x%—percentage of patients in each subgroup given by combination of factors (sum of percentages in each layer = 100%); ITC = isolated tumour cells; None = no micrometastasis, macrometastasis or ITC.

In order to provide a more accurate LNM prediction, we developed a model which takes into account the serum levels of the CA125 and HE4 tumour markers as well as the expected extent of uterine invasion, as determined by ultrasound examination prior to EC verification by biopsy (Figure 3). In the full cohort of 220 patients (100%), LNM was observed in 37 cases (17%). In 178 patients (81%) with serum levels of CA125 < 35 IU/mL, LNM was diagnosed in 11 cases (6%). A total of 42 patients (19%) from the full cohort exhibited serum levels of CA125 ≥ 35 IU/mL. The model classifies these patients as high-risk, as LNM was observed in 27 patients (64%) from this category. Patients with CA125 < 35 IU/mL were further stratified according to serum levels of HE4 and the extent of uterine invasion as determined by ultrasound. Patients with CA125 < 35 IU/mL and HE4 < 142 pmol/L are considered low-risk. In a cohort of 160 patients (73%), LNM was diagnosed in two patients (1%). The model also classifies patients with CA125 < 35 IU/mL and HE4 ≥ 142 pmol/L as low-risk in case no myometrial invasion was observed or in case of tumour invasion limited to <½ of the myometrium. This group includes 7 patients (3%), none of whom were diagnosed with LNM. Patients with CA125 < 35 IU/mL, HE4 ≥ 142 pmol/L, and with an observed tumour invasion of ≥½ of the myometrium, uterine serosal invasion, or cervical stromal invasion, were classified as high-risk. LNM was observed in 9 out of 11 patients (5%) with these characteristics. Patients with CA125 ≥ 35 IU/mL and without myometrial invasion or with tumour invasion limited to <½ of the myometrium included 7 patients (3%), of whom 1 patient (14%) was diagnosed with LNM. Patients with an observed tumour invasion of ≥½ of the myometrium, uterine serosal invasion, or cervical stromal invasion, and simultaneously with HE4 ≥ 103 pmol/L were clearly classified as high-risk. Of the 24 patients (11%) in the group, 21 (88%) were diagnosed with LNM. The model also identified a hard to classify group of 11 patients (5%) with tumour invasion findings identical to the previous group, but with HE4 < 103 pmol/L; 5 of these patients (45%) were diagnosed with LNM. The prediction model comprises 3 investigated parameters (CA125 levels, HE4 levels, and the extent of uterine invasion established by means of ultrasound examination) and yields a sensitivity of 84% (95% CI: 77–89%) and a specificity of 98% (94–99%), and an AUC of 0.95 (95% CI: 0.91–0.99).

## 4. Discussion

To date, no clearly defined tumour markers have been adopted for routine use in EC staging. Such markers should have clearly defined prognostic significance, including sufficiently accurate LNM risk prediction capacity and rational usage in patient follow-up after treatment. This study thus investigated the potential of the serum levels of CA125 and HE4 tumour markers in order to assess their LNM prediction capacity as well as potential use for the risk stratification of regional lymph node involvement. Both CA125 and HE4 typically exhibit a broad range of values in the case of EC which can complicate clinical use [19,26]. Therefore, it is necessary to eliminate factors leading to false positives, define exclusion and inclusion criteria, and improve model performance in combination with the ultrasound assessment of the extent of uterine infiltration. This study found a significant elevation of CA125 and HE4 levels in LNM cases, which is corroborated by the results of previous studies addressing the issue.

We acknowledge regional, design-related, and histopathology-dependent differences between published cohorts and their impact on generalizability. One limitation of this study is the relatively small sample size, which constrained the use of more extensive internal validation techniques such as repeated k-fold cross-validation or bootstrapping. However, 2-fold cross-validation was applied to reduce overfitting. The elevation of CA125 levels in the case of EC has been described in a number of papers where the levels have been correlated with, for example, the depth of tumour invasion into the myometrium, disease stage, and tumour grade [11,27,28,29].

In a retrospective study of 92 patients, Chung et al. hypothesize that serum CA125 level can serve as a potential predictor of LN involvement at a cut-off value of 28.5 IU/mL. The sensitivity and specificity parameters at this cut-off value were 61.5% and 94.9%, respectively. The study focused on the following histological types: endometrioid adenocarcinoma, adenosquamous carcinoma, clear cell carcinoma, and papillary serous carcinoma. All patients underwent surgical LN staging, i.e., pelvic and/or para-aortic lymph node dissection. A total of 13 patients (14.1%) were diagnosed with LNM. Elevated CA125 levels (>28.5 IU/mL) were found to be significantly associated with LNM (*p* < 0.001). The study included patients with comorbidities potentially affecting CA125 levels. No additional tumour markers were included in the study [22].

A prospective multicentric study by Antonsen et al. determined a cut-off value for serum HE4 levels as an LNM risk at 70 pmol/L, with a sensitivity of 75.9% and a specificity of 48.8%. Serum CA125 level as a predictor of LNM yields a sensitivity of 62.1% and specificity of 78.9% at a cut-off of 35 IU/mL. At a cut-off of 20 IU/mL, the parameters yielded a sensitivity of 86.2% and a specificity of 52.8%. The median of both tumour markers was significantly increased in patients with histologically confirmed LNM: CA125 45.5 U/mL (*p* < 0.001) and HE4 141.5 pmol/L (*p* < 0.013). The study identified a significant positive correlation between CA125 and HE4 levels and histological grade, myometrial invasion, and cervical involvement. The study included a total of 352 patients with biopsy-proven EC or atypical hyperplasia (AH). A total of 346 patients underwent surgical procedures; however, it is important to note that only 146 patients (42.2%) underwent lymphadenectomy, which may have resulted in an underestimation of the number of patients with LNM. LNM was diagnosed in 29 patients (8.2% of the entire cohort) [15].

In a retrospective study, Wang et al. reported serum CA125 levels as a predictor for LNM at an achieved cut-off of 13.5 U/mL and HE4 levels at a cut-off of 72.9 pmol/L The sensitivity and specificity values were 72.2% and 51.9% for CA125 and 82.4% and 52.3% for HE4. The study included 258 patients diagnosed with EC without providing additional histological subtype specifications. Exclusion criteria included the initiation of neoadjuvant therapy or hormone therapy. Tumour marker collection was performed one day prior to surgery. Each patient underwent pelvic and para-aortic lymphadenectomy. LNM was diagnosed in 17 patients (6.6%). The combination of CA125 and HE4 cut-off values utilised in this study yielded a superior sensitivity parameter of 94.1%, thus surpassing the sensitivity parameters of CA125 and HE4 cut-off values employed individually [20].

In a retrospective multicentric study, Lombaers et al. established a cut-off value of CA125 as a risk of LNM in HG EC 35 IU/mL, with a sensitivity of 68.4% and a specificity of 78.7%. The study included a total of 333 patients with a HG carcinoma diagnosis, established by endometrial biopsy, whose serum CA125 levels were established prior to surgery. Surgical LN staging was performed as pelvic and/or para-aortic lymphadenectomy in 193 patients (58.1%). LNM was diagnosed in 29.4% of patients who underwent LN staging. An elevated serum CA125 level (>35 IU/mL) was found to be significantly associated with a higher risk of LNM (*p* < 0.001) and FIGO disease stage III–V (*p* < 0.001). The study further established that in patients with normal CA125 levels, preoperative CT with suspected LNM was not significant for LNM prediction. The utilisation of HE4 was not a primary objective of the study [18]. The preoperative elevation of CA125 as an LNM risk factor has been documented in other studies [30,31].

A retrospective study by O’Toole et al. which evaluated the use of CA125 and HE4 levels as a preoperative stratifier for LNM listed a cut-off value for HE4 as 81 pmol/L with a sensitivity of 78.6% and a specificity of 53.4% while the cut-off value for CA125 was established as 35 IU/mL with a sensitivity of 57% and a specificity of 91.4% [21]. In comparison, in our study an identical CA125 cut-off value of 35 IU/mL yielded a sensitivity of 70% and a specificity of 92%. For an HE4 cut-off value of 103 IU/mL, the sensitivity and specificity were 78% and 80%, respectively. A comparison of CA125 and HE4 cut-off levels specified in individual studies is included in Table 4.

The study conducted by the Irish researchers focuses exclusively on patients diagnosed with endometrioid carcinoma. In our study cohort, LNM was detected by surgical staging in 37 patients (16.8%), whereas O’Toole et al. detected LNM in 14 patients (9.5%). The work of O’Toole et al. tracks the correlation of CA125 and HE4 levels with other EC risk factors. Median serum HE4 and CA125 levels were found to be significantly elevated in cases of high-grade carcinomas, in cases with myometrial invasion of > 50%, and in the presence of lymphovascular space invasion (LVSI). The study revealed no correlation between tumour marker elevation and BMI, diabetes, smoking, or alcohol use. In our study, the observed comorbidities did not appear to be significant LNM factors.

As our study adopts a retrospective approach, for statistical analysis the cohort was exclusively composed of patients who underwent sufficient surgical LN staging, followed by histopathology and LN evaluation. The distribution of patients and their characteristics in terms of age, histological tumour type, disease stage, and other parameters is correlated with the general characteristics of patients affected by this disease in middle and high-income countries. In contrast to the studies discussed above, the tumour marker parameters in our cohort were also examined specifically in relation to the nature of metastatic involvement, i.e., micro and macrometastatic. The median CA125 levels were found to be significantly different in cases of micrometastatic and macrometastatic involvement, with median levels of 37 IU/ml and 103 IU/ml, respectively. The extended LNM prediction model, which incorporates three variables: serum CA125 level, serum HE4 level, and the extent of uterine infiltration as determined by preoperative ultrasound examination, yielded sensitivity, and specificity values of 84% and 98%, respectively. The applicability of the model in routine clinical practice is contingent upon the examiners’ proficiency in ultrasonography, which is essential for assessing the extent of uterine infiltration. The validation of findings by comparing ultrasound findings with postoperative histology in terms of uterine infiltration is essential. All ultrasound examinations should be carried out by or under the supervision of licensed examiners according to a standardized protocol [32]. The utilisation of MRI or ultrasound—performed by an expert sonographer—for evaluating myometrial invasion or cervical involvement has been demonstrated to exhibit good and comparable performance metrics and is endorsed in accordance with the ESGO-ESTRO-ESP guideline [5,33,34]. An expert sonographer performs a high volume of gynaecological ultrasounds as their primary focus and is involved in ultrasound-related research and teaching, especially in the field of gynaecological oncology [35]. In conjunction with other models, the extended model proposed here combines serum CA125 and HE4 levels with ultrasound findings describing the extent of uterine infiltration, thus further differentiating LNM risk. Two groups of low-risk patients are thus clearly defined: with CA125 levels < 35 IU/ml and HE4 levels < 142 pmol/l, and with CA125 levels < 35 IU/ml and HE4 levels > 142 pmol/l and a simultaneous absence of ultrasound evidence of myometrial invasion or tumour invasion of < ½ of the myometrium. In view of the minimal LNM risk present in these low-risk patients, surgical LN staging could be avoided in case of severe comorbidities. While patients with CA125 levels ≥ 35 IU/ml and ultrasound findings indicating either no invasion or invasion of < ½ of the myometrium are also classified as low-risk according to the model, this assessment is based on a limited number of patients. On the other hand, a markedly elevated LNM risk, indicating that surgical LN staging is highly appropriate, is relevant to two other patient groups. The first is characterised by CA125 levels of < 35 IU/ml, HE4 levels of ≥142 pmol/l, and the presence of tumour invasion of ≥ ½ of the myometrium, uterine serosal invasion, or cervical stromal invasion, as determined by ultrasound imaging; LNM probability in this group is 80%. Surgical LN staging clearly is indicated in patients with CA125 levels ≥ 35 IU/ml, HE4 levels ≥ 103 pmol/l, and the presence of tumour invasion of ≥ ½ of the myometrium, uterine serosal invasion, or cervical stromal invasion; LNM probability in this group is 88%. The findings of this study demonstrate that CA125 and HE4 may be utilised as markers in future prediction models integrating information on serum tumour marker levels with the results of accessible and cost-effective imaging methods capable of describing the extent of uterine infiltration. In accordance with the recommendations of ESGO and ESTRO, imaging techniques play a pivotal role in the assessment of disease extent, with MRI and PET/CT exhibiting high specificity in the evaluation of lymph node involvement [36,37,38].

In practice, however, the number of patients and imaging-related expenses necessitates a simple and cost-effective marker or model with a high sensitivity and specificity. Such a model should be available at the primary care stage and should include some degree of personalisation, incorporating individual patient characteristics and serum levels of laboratory markers. Such a model would also be beneficial in medical systems with a reduced availability or resources for imaging modalities such as CT, MRI, or hybrid imaging modalities.

The CA125 and HE4 cut-off values and the developed model demonstrate sufficient potential for utilisation in patient stratification, with a favourable impact on clinical practice, i.e., the potential possibility of reducing the extent of surgery in patients compromised by significant internal comorbidities while eliminating the risk of adverse downstaging. In clinical practice, the omission of completion lymphadenectomy is currently an accepted approach in cases of low-risk disease with failure of SNB [5].

A fundamental prerequisite for introducing the developed model into clinical practice is cooperation between members of a certified oncogynaecological team, including an expert sonographer, pathologist, and certified laboratory department. This would naturally follow model validation in further prospective multicentric studies with identical designs. Further improvements to our model or similar models would include integrating molecular classification or constructing defined nomograms.

## 5. Conclusions

The results of our study suggest that preoperative CA125 and HE4 levels, as well as the developed model, could potentially be used to determine the extent of surgery. Currently, lymph node mapping and sampling are standard practice and should be performed during surgery, while bearing in mind that omitting completion lymphadenectomy is already an accepted approach in case of low-risk disease. Our model might be validated to be used to determine whether complete lymphadenectomy is warranted or could be omitted in patients who fail to map. The resulting benefit for clinical practice is the possibility of reducing the extent of surgery in patients compromised by significant internal comorbidities, while eliminating the risk of undesirable downstaging. We are aware of the limitations of our study, i.e., no external validation and a retrospective and single-centric design. Further validation based on multicentric studies of an identical design is required to confirm these results. In order to develop a more complete understanding of the potential of our model or similar models in the future, it is necessary to consider integration with genomic classifiers and validation in low-resource/low-imaging environments.

## Figures and Tables

**Table 1:** Population characteristics.

Characteristic	N = 220
**Age (years)**	
Mean (SD)	64 (10)
Median (IQR)	65 (59–70)
**Metastatic involvement** ***, n (%)***	
No	167 (75.9%)
ITC	16 (7.3%)
Micrometastasis	13 (5.9%)
Macrometastasis	24 (10.9%)
**CA125 (IU/mL)**	
Mean (SD)	38 (88)
Median (IQR)	16 (10–29)
**HE4 (pmol/L)**	
Mean (SD)	110 (142)
Median (IQR)	78 (59–109)
**Histotype** ***, n (%)***	
Endometrioid LG	159 (72.3%)
Endometrioid HG	34 (15.5%)
Serous	16 (7.3%)
Mixed with endometrioid component	11 (5.0%)
**Surgical staging** ***, n (%)***	
Bilateral SNB detection	158 (71.8%)
Unilateral SNB + systemic PLN contralaterally	6 (2.7%)
Systemic PLN + PALN	53 (24.1%)
Bulky lymph node resection	3 (1.4%)
**Tumour invasion** ***, n (%)***	
Limited to endometrium	35 (15.9%)
Invasion of <½ myometrium	88 (40.0%)
Invasion of ≥½ myometrium	47 (21.4%)
Infiltration of cervical stroma	41 (18.6%)
Infiltration of uterine serosa	9 (4.1%)
**FIGO** ***, n (%)***	
IA	119 (54.1%)
IB	28 (12.7%)
II	29 (13.2%)
IIIA	6 (2.7%)
IIIB	1 (0.5%)
IIIC1	27 (12.3%)
IIIC2	8 (3.6%)
IVA	0 (0.0%)
IVB	2 (0.9%)
**FIGO stage** ***, n (%)***	
Local disease (stage I + II)	176 (80.0%)
Advanced disease (stage III + IV)	44 (20.0%)
**Obesity** ***, n (%)***	
No	94 (42.7%)
Yes	126 (57.3%)
**Hypertension** ***, n (%)***	
No	86 (39.1%)
Yes	134 (60.9%)
**IHD** ***, n (%)***	
No	205 (93.2%)
Yes	15 (6.8%)
**Diabetes mellitus** ***, n (%)***	
No	171 (77.7%)
Yes	49 (22.3%)

SD = standard deviation; IQR = interquartile range; ITC = isolated tumour cells; LG = low grade; HG = high grade; SNB = sentinel lymph node biopsy; PLN = pelvic lymphadenectomy; PALN = para-aortic lymphadenectomy; IHD = ischemic heart disease; FIGO = International Federation of Gynaecology and Obstetrics.

**Table 2:** Population characteristics according to metastatic involvement.

Characteristic	None N = 167	Micrometastasis N = 13	Macrometastasis N = 24	ITC N = 16	*p*-Value ^1^
**Age (years)**					**0.041**
Mean (SD)	**63** (10)	65 (13)	67 (5)	68 (8)	
Median (IQR)	65 (58–69)	62 (54–79)	69 (64–70)	68 (65–73)	
**CA125 (IU/mL)**					**<0.001**
Mean (SD)	19 (19)	70 (86)	153 (219)	39 (54)	
Median (IQR)	14 (9–21)	37 (35–65)	103 (28–158)	26 (16–31)	
**HE4 (pmol/L)**					**<0.001**
Mean (SD)	81 (45)	153 (75)	276 (367)	121 (75)	
Median (IQR)	73 (55–93)	144 (105–179)	160 (118–255)	81 (76–120)	
**Histotype** ***, n (%)***					
Endometrioid LG	126 (75.4%)	10 (76.9%)	9 (37.5%)	14 (87.5%)	
Endometrioid HG	22 (13.2%)	2 (15.4%)	9 (37.5%)	1 (6.2%)	
Serous	11 (6.6%)	1 (7.7%)	4 (16.7%)	0 (0.0%)	
Mixed with endometroid component	8 (4.8%)	0 (0.0%)	2 (8.3%)	1 (6.2%)	
**Surgical staging** ***, n (%)***					**<0.001**
Bilateral SNB detection	125 (74.9%)	13 (100.0%)	7 (29.2%)	13 (81.2%)	
Unilateral SNB + systemic PLN contralaterally	3 (1.8%)	0 (0.0%)	2 (8.3%)	1 (6.2%)	
Systemic PLN + PALN	38 (22.8%)	0 (0.0%)	14 (58.3%)	1 (6.2%)	
Bulky lymph node resection	1 (0.6%)	0 (0.0%)	1 (4.2%)	1 (6.2%)	
**Tumour invasion** ***, n (%)***					
Limited to endometrium	35 (21.0%)	0 (0.0%)	0 (0.0%)	0 (0.0%)	
Invasion of <½ myometrium	78 (46.7%)	0 (0.0%)	1 (4.2%)	9 (56.2%)	
Invasion of ≥½ myometrium	26 (15.6%)	10 (76.9%)	8 (33.3%)	3 (18.8%)	
Infiltration of cervical stroma	27 (16.2%)	3 (23.1%)	7 (29.2%)	4 (25.0%)	
Infiltration of uterine serosa	1 (0.6%)	0 (0.0%)	8 (33.3%)	0 (0.0%)	
**FIGO** ***, n (%)***					
IA	110 (65.9%)	0 (0.0%)	0 (0.0%)	9 (56.2%)	
IB	26 (15.6%)	0 (0.0%)	0 (0.0%)	2 (12.5%)	
II	25 (15.0%)	0 (0.0%)	0 (0.0%)	4 (25.0%)	
IIIA	6 (3.6%)	0 (0.0%)	0 (0.0%)	0 (0.0%)	
IIIB	0 (0.0%)	0 (0.0%)	0 (0.0%)	1 (6.2%)	
IIIC1	0 (0.0%)	13 (100.0%)	14 (58.3%)	0 (0.0%)	
IIIC2	0 (0.0%)	0 (0.0%)	8 (33.3%)	0 (0.0%)	
IVA	0 (0.0%)	0 (0.0%)	0 (0.0%)	0 (0.0%)	
IVB	0 (0.0%)	0 (0.0%)	2 (8.3%)	0 (0.0%)	
**FIGO stage** ***, n (%)***					**<0.001**
Local disease	161 (96.4%)	0 (0.0%)	0 (0.0%)	15 (93.8%)	
Advanced disease	6 (3.6%)	13 (100.0%)	24 (100.0%)	1 (6.2%)	
**Obesity** ***, n (%)***					0.177
No	70 (41.9%)	8 (61.5%)	7 (29.2%)	9 (56.2%)	
Yes	97 (58.1%)	5 (38.5%)	17 (70.8%)	7 (43.8%)	
**Hypertension** ***, n (%)***					0.506
No	68 (40.7%)	5 (38.5%)	6 (25.0%)	7 (43.8%)	
Yes	99 (59.3%)	8 (61.5%)	18 (75.0%)	9 (56.2%)	
**IHD** ***, n (%)***					0.537
No	157 (94.0%)	12 (92.3%)	22 (91.7%)	14 (87.5%)	
Yes	10 (6.0%)	1 (7.7%)	2 (8.3%)	2 (12.5%)	
**Diabetes mellitus** ***, n (%)***					0.146
No	131 (78.4%)	10 (76.9%)	21 (87.5%)	9 (56.2%)	
Yes	36 (21.6%)	3 (23.1%)	3 (12.5%)	7 (43.8%)	

^1^ Kruskal–Wallis rank sum test; Fisher’s exact test; Pearson’s Chi-squared test; bolded values indicate statistical significance (*p* < 0.05); ITC = isolated tumour cells; SD = standard deviation; IQR = interquartile range; LG = low grade; HG = high grade; SNB = sentinel lymph node biopsy; PLN = pelvic lymphadenectomy; PALN = para-aortic lymphadenectomy; IHD = Ischemic Heart Disease; FIGO = International Federation of Gynaecology and Obstetrics.

**Table 3:** Population characteristics of two prognostically different groups.

Characteristic	None + ITC, N = 183	Micro-/Macrometastasis, N = 37	*p*-Value ^1^
**Age (years)**			0.086
Mean (SD)	64 (10)	67 (9)	
Median (IQR)	65 (58–70)	68 (62–71)	
**Metastatic involvement** ***, n (%)***			**<0.001**
No	167 (91.3%)	0 (0.0%)	
ITC	16 (8.7%)	0 (0.0%)	
Micrometastasis	0 (0.0%)	13 (35.1%)	
Macrometastasis	0 (0.0%)	24 (64.9%)	
**CA125 (IU/mL)**			**<0.001**
Mean (SD)	21 (24)	124 (187)	
Median (IQR)	14 (9–23)	64 (30–125)	
**HE4 (pmol/L)**			**<0.001**
Mean (SD)	85 (49)	233 (303)	
Median (IQR)	74 (56–96)	154 (105–225)	
**Histotype** ***, n (%)***			**0.010**
Endometrioid LG	140 (76.5%)	19 (51.4%)	
Endometrioid HG	23 (12.6%)	11 (29.7%)	
Serous	11 (6.0%)	5 (13.5%)	
Mixed with endometroid component	9 (4.9%)	2 (5.4%)	
**Surgical staging** ***, n (%)***			**0.035**
Bilateral SNB detection	138 (75.4%)	20 (54.1%)	
Unilateral SNB + systemic PLN contralaterally	4 (2.2%)	2 (5.4%)	
Systemic PLN + PALN	39 (21.3%)	14 (37.8%)	
Bulky lymph nodes resection	2 (1.1%)	1 (2.7%)	
**Tumour invasion** ***, n (%)***			**<0.001**
Limited to endometrium	35 (19.1%)	0 (0.0%)	
Invasion of <½ myometrium	87 (47.5%)	1 (2.7%)	
Invasion of ≥½ myometrium	29 (15.8%)	18 (48.6%)	
Infiltration of cervical stroma	31 (16.9%)	10 (27.0%)	
Infiltration of uterine serosa	1 (0.5%)	8 (21.6%)	
**FIGO** ***, n (%)***			**<0.001**
IA	119 (65.0%)	0 (0.0%)	
IB	28 (15.3%)	0 (0.0%)	
II	29 (15.8%)	0 (0.0%)	
IIIA	6 (3.3%)	0 (0.0%)	
IIIB	1 (0.5%)	0 (0.0%)	
IIIC1	0 (0.0%)	27 (73.0%)	
IIIC2	0 (0.0%)	8 (21.6%)	
IVA	0 (0.0%)	0 (0.0%)	
IVB	0 (0.0%)	2 (5.4%)	
**FIGO stage** ***, n (%)***			**<0.001**
Local disease	176 (96.2%)	0 (0.0%)	
Advanced disease	7 (3.8%)	37 (100.0%)	
**Obesity** ***, n (%)***			0.768
No	79 (43.2%)	15 (40.5%)	
Yes	104 (56.8%)	22 (59.5%)	
**Hypertension** ***, n (%)***			0.201
No	75 (41.0%)	11 (29.7%)	
Yes	108 (59.0%)	26 (70.3%)	
**IHD** ***, n (%)***			0.722
No	171 (93.4%)	34 (91.9%)	
Yes	12 (6.6%)	3 (8.1%)	
**Diabetes mellitus** ***, n (%)***			0.332
No	140 (76.5%)	31 (83.8%)	
Yes	43 (23.5%)	6 (16.2%)	

^1^ Mann–Whitney test; Fisher’s exact test; Pearson’s Chi-squared test; bolded values indicate statistical significance (*p* < 0.05); ITC = isolated tumour cells; SD = standard deviation; IQR = interquartile range; LG = low grade; HG = high grade; SNB = sentinel lymph node biopsy; PLN = pelvic lymphadenectomy; PALN = para-aortic lymphadenectomy; IHD = Ischemic Heart Disease; FIGO = International Federation of Gynaecology and Obstetrics.

**Table 4:** Summary and characteristics of recent studies evaluating cancer antigen 125 (CA125) and human epididymis protein 4 (HE4) cut-off values for lymph node metastasis (LNM).

Study	Year	CA125 Cut-Off (IU/mL)	Sensitivity (%)	Specificity (%)	HE4 Cut-Off (pmol/L)	Sensitivity (%)	Specificity (%)	Study Design	Sample Size
Antonsen et al. [15]	2013	35	62.1	78.9	70	75.8	48.4	Prospective	352
Wang et al. [20]	2016	13.5	72.2	51.9	72.9	82.4	52.3	Retrospective	258
O’Toole et al. [21]	2020	35	57	91.4	81	78.6	53.4	Retrospective	147
Chung et al. [22]	2011	28.5	61.5	94.9	–	–	–	Retrospective	92
Lombaers et al. [18]	2023	35	68.4	78.7	–	–	–	Retrospective	333

## Data Availability

Data are available upon reasonable request.
